# Supplementary material for: Characterization of the NRAMP Gene Family in the Arbuscular Mycorrhizal Fungus Rhizophagus irregularis
Source: J Fungi (Basel). 2022 May 31;8(6):592. doi: 10.3390/jof8060592 (PMC9224570; doi:10.3390/jof8060592)
Supplement: Supplementary file 1 [file jof-08-00592-s001.zip › jof-1696479-supplementary.pdf]

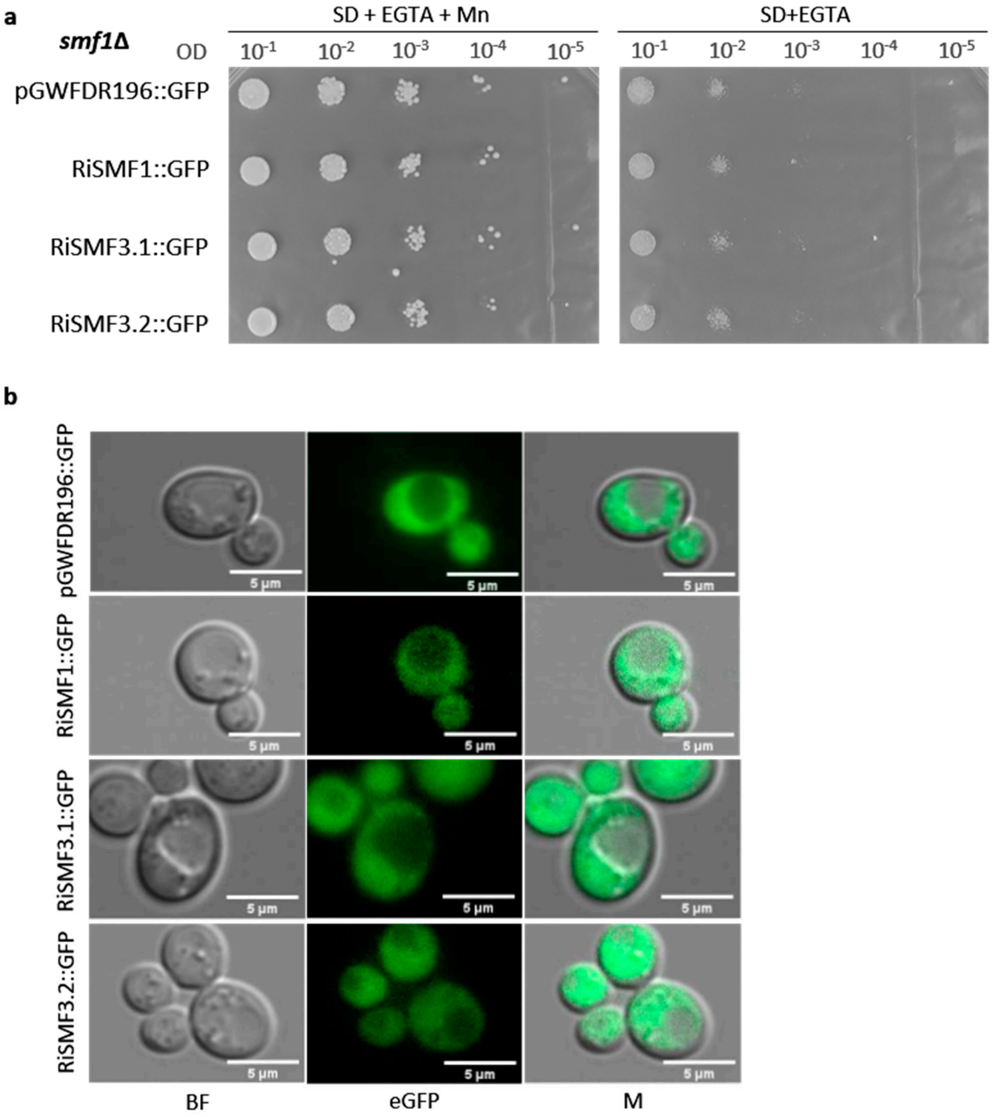

Figure S1. (a) *smf1*Δ yeast cells expressing GFP (empty vector pGWFDR19) or C-terminal GFP-tagged version of RiSMF1, RiSMF3.1 or RiSMF3.2 were plated on SD-URA media with 50 mM MES buffer pH 6, 6.25 mM EGTA and supplemented or not with 500 µM $MnSO_4$. The cultures were diluted to ODs of 10^{-1} to 10^{-5} (as indicated) and spotted on the corresponding selective media. (b) *smf1*Δ cells expressing GFP (empty vector pGWFDR196) and C-terminal GFP-tagged versions of *RiSMF1*, *RiSMF3.1* or *RiSMF3.2* were visualized in a fluorescence microscope. BF, bright field; eGFP, enhanced GFP fluorescence; M, merged images.

Table S1. Oligonucleotides used in this study. Overhangs are underlined

Primer	Sequence (5'-3')[a]	Application
**qRiSMF1.F**	GGGTGTCGTTACAGGAATGG	Real Time PCR
**qRiSMF1.R**	TGCAACTCCCCAAGGTAAAG	Real Time PCR
**qRiSMF2.F**	TAGCGCAAGCATGTAAAGCA	Real Time PCR
**qRiSMF2.R**	TTTAATCCCATCGCACTTCC	Real Time PCR
**qRiSMF3.1.F**	AACGAAGATAGGCCTTTACCA	Real Time PCR
**qRiSMF3.1.R**	CCGTGATGCATCTCCCAAG	Real Time PCR
**qRiSMF3.2.F**	CCACTTGATGGAAAACAAGAA	Real Time PCR
**qRiSMF3.2.R**	CGAAGAATTTTTATCCCGAATG	Real Time PCR
**qRiEF1αF**	GCTATTTTGATCATTGCCGCC	Real Time PCR
**qRiEF1αR**	TCATTAAAACGTTCTTCCGACC	Real Time PCR
**qCiEF1αF**	CATGCGTCAGACGGTTGCTGT	Real Time PCR
**qCiEF1αR**	CTTCACTCCCTTCTTGGCTGC	Real Time PCR
**RiSMF1.GW5**	AAAGCAGGCTTCATGAATTATCCGACAAAAGA	Cloning RiSMF1
**RiSMF1.GW3**	GAAAGCTGGGTCTTACAAAGTACCTTCAATCG	Cloning RiSMF1
**RiSMF1.GW3gfp**	GAAAGCTGGGTCTTCCAAAGTACCTTCAATCG	Cloning RiSMF1 with STOP codon modified
**RiSMF3.1.GW5**	AAAGCAGGCTTCATGCAACAAGGGAAAATAGT	Cloning RiSMF3.1
**RiSMF3.1.GW3**	GAAAGCTGGGTCTCAATCTGAGATATTATTAA	Cloning RiSMF3.1
**RiSMF3.1.GW3gfp**	GAAAGCTGGGTCTTCATCTGAGATATTATTAA	Cloning RiSMF3.1 with STOP codon modified
**RiSMF3.2.GW5**	AAAGCAGGCTTCATGCAACAAGGGAAAATAGT	Cloning RiSMF3.2
**RiSMF3.2.GW3**	GAAAGCTGGGTCTTATCCCGAATGATTTGTCA	Cloning RiSMF3.2
**RiSMF3.2.GW3gfp**	GAAAGCTGGGTCTTCTCCCGAATGATTTGTCA	Cloning RiSMF3.2 with STOP codon modified

Table S2. Percent similarity matrix of NRAMP proteins from *Rhizophagus irregularis* (Ri), *Saccharomyces cerevisiae* (Sc), *Paenibacillus sp.* (Ps), *Arabidopsis thaliana* (At) and *Caenorhabditis elegans* (Ce).

	**RiSMF1**	**RiSMF2**	**RiSMF3.1**	**RiSMF3.2**
**RiSMF1**	100	45	33	32
**RiSMF2**	45	100	28	30
**RiSMF3.1**	32	28	100	55
**RiSMF3.2**	32	30	55	100
**ScSMF1**	38	34	28	29
**ScSMF2**	38	36	27	29
**ScSMF3**	36	34	27	26
**AtNRAMP3**	31	29	39	41
**AtNRAMP4**	31	29	39	42
**Ps WP_193723414.1**	40	39	30	32
**Ce WP_040990443.1**	30	29	40	41
